# Regulation of substituent groups on morphologies and self-assembly of organogels based on some azobenzene imide derivatives

**DOI:** 10.1186/1556-276X-8-160

**Published:** 2013-04-08

**Authors:** Tifeng Jiao, Yujin Wang, Qingrui Zhang, Jingxin Zhou, Faming Gao

**Affiliations:** 1 Hebei Key Laboratory of Applied Chemistry, School of Environmental and Chemical Engineering, Yanshan University, Qinhuangdao 066004, China; 2 State Key Laboratory of Solid Lubrication, Lanzhou Institute of Chemical Physics, Chinese Academy of Sciences, Lanzhou 730000, China

**Keywords:** Organogel, Nanostructures, Self-assembly, Substituent groups, Imide derivative, Azobenzene

## Abstract

In this paper, new azobenzene imide derivatives with different substituent groups were designed and synthesized. Their gelation behaviors in 21 solvents were tested as novel low-molecular-mass organic gelators. It was shown that the alkyl substituent chains and headgroups of azobenzene residues in gelators played a crucial role in the gelation behavior of all compounds in various organic solvents. More alkyl chains in molecular skeletons in present gelators are favorable for the gelation of organic solvents. Scanning electron microscopy and atomic force microscopy observations revealed that the gelator molecules self-assemble into different aggregates, from wrinkle, lamella, and belt to fiber with the change of solvents. Spectral studies indicated that there existed different H-bond formations between amide groups and conformations of methyl chains. The present work may give some insight to the design and character of new organogelators and soft materials with special molecular structures.

## Background

It is well known that organogels are one class of important soft materials, in which organic solvents are immobilized by gelators [1-6]. Although gels are widely found in polymer systems, there has recently been an increasing interest in low-molecular-mass organic gelators (LMOGs) [7,8]. In recent years, physical gelation of organic solvents by LMOGs has become one of the hot areas in the soft matter research due to their scientific values and many potential applications in the biomedical field, including tissue engineering, controlled drug release, medical implants, and so on [9-14]. The gels based on LMOGs are usually considered as supramolecular gels, in which the gelator molecules self-assemble into three-dimensional networks in which the solvent is trapped via various non-covalent interactions, such as hydrogen bonding, π-π stacking, van der Waals interaction, dipole-dipole interaction, coordination, solvophobic interaction, and host-guest interaction [15-20]. Such organogels have some advantages over polymer gels: the molecular structure of the gelator is defined, and the gel process is usually reversible. Such properties make it possible to design various functional gel systems and produce more complicated and defined, as well as controllable, nanostructures [21-25].

In our reported work, the gelation properties of some cholesterol imide derivatives consisting of cholesteryl units and photoresponsive azobenzene substituent groups have been investigated [26]. We found that a subtle change in the headgroup of azobenzene segment can produce a dramatic change in the gelation behavior of both compounds. In addition, the gelation properties of bolaform and trigonal cholesteryl derivatives with different aromatic spacers have been characterized [27]. Therein, we have investigated the spacer effect on the microstructures of such organogels and found that various kinds of hydrogen bond interactions among the molecules play an important role in the formation of gels.

As a continuous work, herein, we have designed and synthesized new azobenzene imide derivatives with different substituent groups. In all compounds, the long alkyl chains were symmetrically attached to a benzene ring to form single or three substituent states, with the azobenzene as substituent headgroups. We have found that all compounds could form different organogels in various organic solvents. Characterization of the organogels by scanning electron microscopy (SEM) and atomic force microscopy (AFM) revealed different structures of the aggregates in the gels. We have investigated the effect of alkyl substituent chains and headgroups of azobenzene residues in gelators on the microstructures of such organogels in detail and found different kinds of hydrogen bond interactions between amide groups and conformations of methyl chains.

## Methods

### Materials

**Figure 1:**
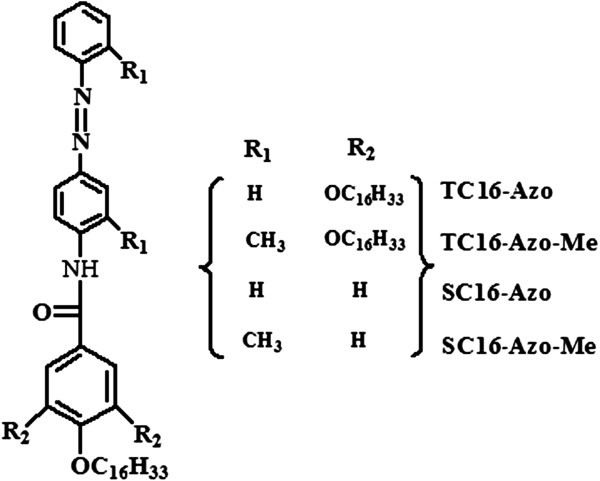
Structures and abbreviations of azobenzene imide derivatives with different substituent groups.

The starting materials, 4-aminoazobenzene and 2-aminoazotoluene were purchased from TCI Development Co., Ltd, Shanghai, China. Other used reagents were all for the analysis purity from either Alfa Aesar (Beijing, China) or Sigma-Aldrich (Shanghai, China) Chemicals. The solvents were obtained from Beijing Chemicals and were distilled before use. Deionized water was used in all cases. 4-Hexadecyloxybenzoic acid and 3,4,5-tris(hexadecyloxy)benzoic acid were synthesized in our laboratory according to a previous report [28] and confirmed by proton nuclear magnetic resonance (^1^H NMR). Then, these azobenzene imide derivatives were prepared by simple methods. Simply speaking, different benzoic acid chlorides were synthesized by heating acid compound solutions in sulfoxide chloride and a bit of dimethylformamide (DMF) for about 10 h at 70°C. Then, the prepared benzoic acid chlorides reacted with the corresponding azobenzene amines in dried dichloromethane at the presence of pyridine for 2 days at room temperature. After that, the mixtures were washed with diluted hydrochloric acid and pure water. The organic layer was evaporated to dryness. The residues were purified by recrystallization in ethanol solution as a yellow solid. The final products and their abbreviations are shown in Figure 1, which were confirmed by ^1^H NMR and elemental analysis.

### Gelation test

A weighted amount of gelator and a measured volume of selected pure organic solvent were placed into a sealed glass bottle, and the solution was heated in a water bath until the solid was dissolved. Then, the solution was cooled to room temperature in air and the test bottle was inversed to see if a gel was formed. When the gelator formed a gel by immobilizing the solvent at this stage, it was denoted as ‘G’. For the systems in which only solution remained until the end of the tests, they were referred to as solution (S). The system in which the potential gelator could not be dissolved, even at the boiling point of the solvent, was designated as an insoluble system (I). Critical gelation concentration (CGC) refers to the minimum concentration of the gelator for gel formation.

### Measurements

Firstly, the xerogel was prepared by a vacuum pump for 12 to 24 h. The dried sample thus obtained was attached to mica, copper foil, glass, and CaF_2_ slice for morphological and spectral investigations. Before SEM measurement, the samples were coated with copper foil fixed by a conductive adhesive tape and shielded with gold. SEM pictures of the xerogel were taken using a Hitachi S-4800 field emission scanning electron microscope (Chiyoda-ku, Japan) with the accelerating voltage of 5 to 15 kV. AFM images were recorded using a multimode 8 scanning probe microscope (Veeco Instrument, Plainview, NY, USA) with silicon cantilever probes. All AFM images were shown in the height mode without any image processing except flattening. Transmission Fourier transform infrared (FT-IR) spectra of the xerogel were obtained using a Nicolet iS10 FT-IR spectrophotometer from Thermo Fisher Scientific Inc. (Waltham, MA, USA) with an average of 32 scans and at a resolution of 4 cm^-1^. The X-ray diffraction (XRD) measurement was conducted using a Rigaku D/max 2550PC diffractometer (Rigaku Inc., Tokyo, Japan). The XRD pattern was obtained using CuKα radiation with an incident wavelength of 0.1542 nm under a voltage of 40 kV and a current of 200 mA. The scan rate was 0.5° min^-1^. ^1^H NMR spectra were obtained using a Bruker ARX-400 NMR spectrometer (Bruker, Inc., Switzerland) in CDCl_3_ with tetramethylsilane (TMS) as an internal standard. The elemental analysis was carried out with the Flash EA Carlo-Erba-1106 Thermo-Quest (Milan, Italy).

## Results and discussion

**Figure 2:**
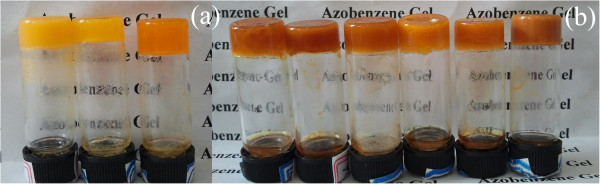
Photographs of organogels of SC16-Azo (a) and SC16-Azo-Me (b) in different solvents.

The gelation performances of all compounds in 21 solvents are listed in Table 1. Examination of the table reveals that all compounds are efficient gelators. Firstly, TC16-Azo can gel in 12 solvents, such as nitrobenzene, aniline, acetone, cyclopentanone, ethyl acetate, pyridine, and DMF. As for TC16-Azo-Me with additional methyl groups in azobenzene part, only eight kinds of organogels were formed. Secondly, as for the SC16-Azo and SC16-Azo-Me with single alkyl substituent chains in molecular skeletons, the numbers of formed organogels changed to 3 and 6, respectively. Their photographs of organogels of SC16-Azo and SC16-Azo-Me in different solvents were shown in Figure 2. The data shown in Table 1 indicate that change of substituent groups in azobenzene residue and benzoic acid derivatives can have a profound effect upon the gelation abilities of these studied compounds. It seemed that more alkyl chains in molecular skeletons in present gelators are favorable for the gelation of organic solvents. In addition, the space effect of methyl groups for intermolecular stacking in the gel formation process is also obvious for all cases. Moreover, in most cases, for a given solvent, the minimum concentration of the gelator for gel formation, named as CGC, is an important factor for the prepared gels [29,30]. In the present case, all compounds can form organogels in DMF. And the CGC values for TC16-Azo and TC16-Azo-Me with three alkyl substituent chains in molecular skeletons seemed smaller than those of compounds with single alkyl substituent chains. The reasons for the strengthening of the gelation behaviors can be assigned to the change of the spatial conformation of the gelators due to the more alkyl substituent chains in molecular skeletons, which may increase the ability of the gelator molecules to self-assemble into ordered structures, a necessity for forming organized network structures.

**Table 1:** Gelation properties of four compounds at room temperature

**Solvents**	**TC16-Azo**	**TC16-Azo-Me**	**SC16-Azo**	**SC16-Azo-Me**
Chloroform	S	S	S	I
Tetrachloromethane	S	S	I	G (4.0)
Benzene	S	S	G (2.0)	G (2.0)
Toluene	S	S	I	I
Nitrobenzene	G (1.5)	G (2.0)	I	G (2.0)
Aniline	G (1.5)	G (2.0)	I	G (2.0)
Acetone	G (1.5)	G (3.0)	I	I
Cyclopentanone	G (1.5)	S	I	I
Cyclohexanone	S	S	I	I
Ethyl acetate	G (2.5)	G (2.0)	I	I
n-Butyl acrylate	S	S	I	I
Petroleum ether	I	I	I	I
Pyridine	G (1.5)	S	G (2.0)	I
DMF	G (1.5)	G (2.0)	G (2.0)	G (3.0)
Ethanol	G (1.5)	I	I	I
n-Propanol	G (2.5)	G (2.0)	I	I
n-Butanol	G (2.5)	G (2.0)	I	I
n-Pentanol	G (2.5)	G (2.0)	I	I
1,4-Dioxane	G (2.5)	S	I	G (3.0)
THF	S	S	I	I
n-Hexane	I	I	I	I

DMF, dimethylformamide; THF, tetrahydrofuran; S, solution; G, gel; I, insoluble; for gels, the critical gelation concentrations at room temperature are shown in parentheses (% w/v).

**Figure 3:**
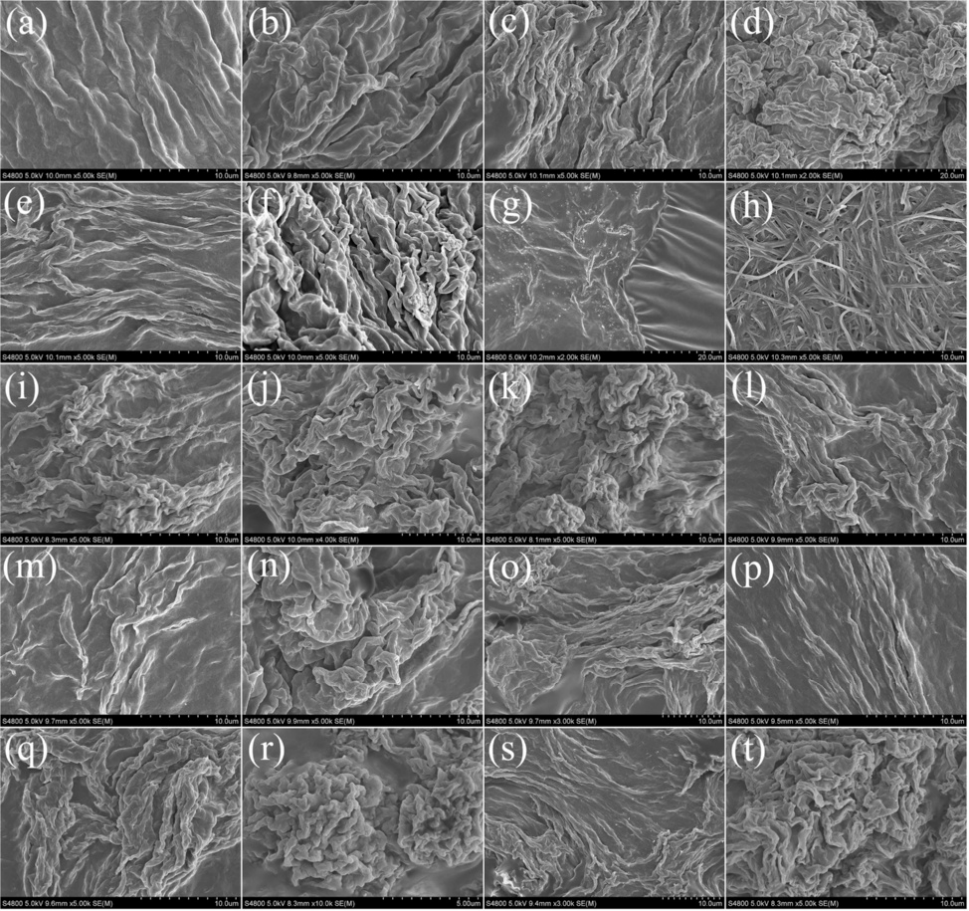
**SEM images of xerogels. **TC16-Azo gels ((**a**) nitrobenzene, (**b**) aniline, (**c**) acetone, (**d**) cyclopentanone, (**e**) ethyl acetate, (**f**) pyridine, (**g**) DMF, (**h**) ethanol, (**i**) n-propanol, (**j**) n-butanol, (**k**) n-pentanol, and (**l**) 1,4-dioxane) and TC16-Azo-Me gels ((**m**) nitrobenzene, (**n**) aniline, (**o**) acetone, (**p**) ethyl acetate, (**q**) DMF, (**r**) n-propanol, (**s**) n-butanol, and (**t**) n-pentanol).

**Figure 4:**
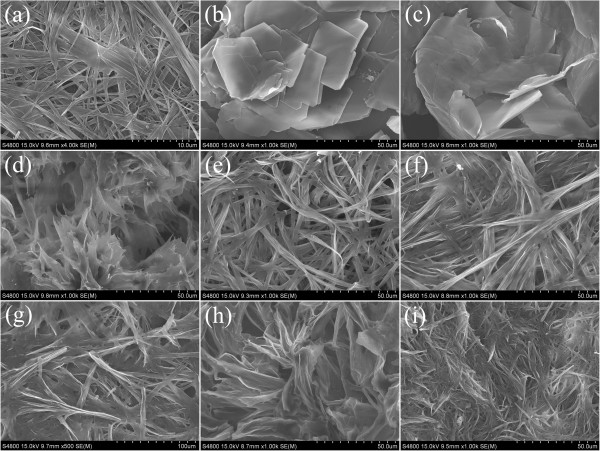
**SEM images of xerogels. **SC16-Azo gels ((**a**) benzene, (**b**) pyridine, and (**c**) DMF) and SC16-Azo-Me gels ((**d**) tetrachloromethane, (**e**) benzene, (**f**) nitrobenzene, (**g**) aniline, (**h**) DMF, and (**i**) 1,4-dioxane).

**Figure 5:**
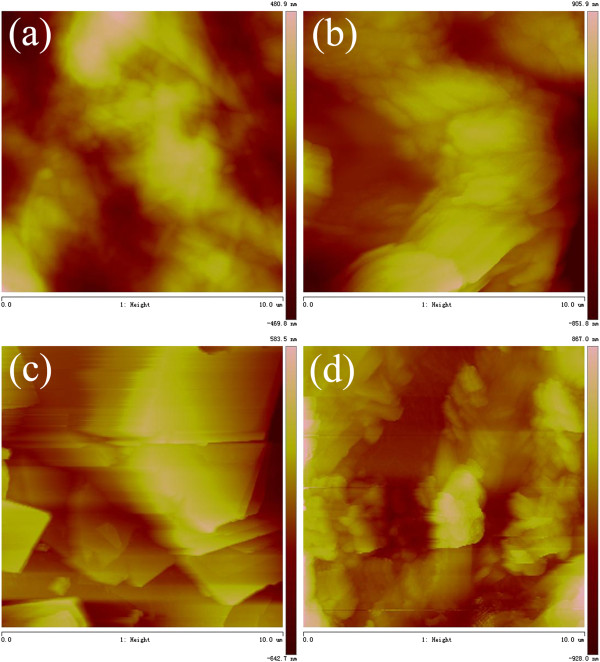
**AFM images of xerogels. **(**a**)TC16-Azo, (**b**) TC16-Azo-Me, (**c**) SC16-Azo, and (**d**) SC16-Azo-Me gels in DMF.

In addition, in order to obtain a visual insight into the gel microstructures, the typical nanostructures of the xerogels were studied using the SEM technique, as shown in Figures 3 and 4. From the present diverse images, it can be easily investigated that the microstructures of the xerogels of all compounds in different solvents are significantly different from each other, and the morphologies of the aggregates change, from wrinkle, lamella, and belt to fiber with the change of solvents. In addition, more regular lamella-like or fiber-like aggregates with different aspect ratios were prepared in the gels of SC16-Azo and SC16-Azo-Me with single alkyl substituent chains in molecular skeletons. As for the two other compounds with multialkyl substituent chains, most of the aggregates tended to have wrinkled or deformed films. Furthermore, the xerogels in DMF of all compounds were characterized by AFM, as shown in Figure 5. From the images, it is interesting to note that these big belt or lamella aggregates were composed of many little domains by the stacking of the present imide derivatives. The morphologies of the aggregates shown in the SEM and AFM images may be rationalized by considering a commonly accepted idea that highly directional intermolecular interactions, such as hydrogen bonding or π-π interactions, favor formation of belt or fiber structures [31-34]. The difference of morphologies between molecules with single alkyl substituent chains and multichains can be mainly due to the different strengths of the intermolecular hydrophobic force between alkyl substituent chains, which have played an important role in regulating the intermolecular orderly staking and formation of special aggregates.

**Figure 6:**
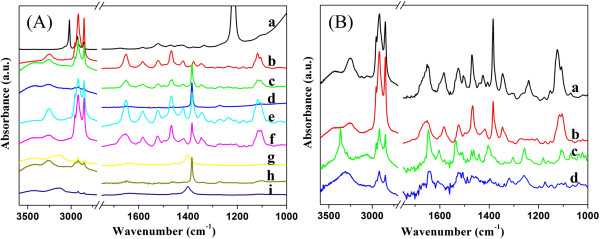
**FT-IR spectra of xerogels. **(**A**) TC16-Azo-Me (**a**, chloroform solution; **b**, nitrobenzene; **c**, aniline; **d**, acetone; **e**, ethyl acetate; **f**, DMF; **g**, n-propanol; **h**, n-butanol; and **i**, n-pentanol); (**B**) **a**, TC16-Azo; **b**, TC16-Azo-Me; **c**, SC16-Azo; and **d**, SC16-Azo-Me, in DMF.

It is well known that hydrogen bonding plays an important role in the formation of organogels [35,36]. At present, in order to further clarify this and investigate the effect of substituent groups on assembly, we have measured the FT-IR spectra of all compounds in chloroform solution and xerogel forms. Firstly, TC16-Azo-Me was taken as an example, as shown in Figure 6A. As for the spectrum of TC16-Azo-Me in chloroform solution, some main peaks were observed at 3,412, 2,926, 2,854, and 1,676 cm^-1^. These bands can be assigned to the N-H stretching, methylene stretching, and the amide I band [37,38]. As far as the spectra of these xerogels, these bands shifted to 3,252, 2,918, 2,848, and 1,651 cm^-1^, respectively. The shift of these bands indicates H-bond formation between amide groups and conformational distortion of methyl chains in the gel state. In addition, the spectra of xerogels of all compounds in DMF were compared, as shown in Figure 6B. One obvious change is the decrement of methylene stretching for SC16-Azo and SC16-Azo-Me in comparison with the other two compounds, which can be attributed to the number difference of alkyl substituent chains in molecular skeletons. Another change is that the peaks assigned to N-H stretching and amide I band for SC16-Azo and SC16-Azo-Me shifted to 3,365, 3,310, and 1,645 cm^-1^, respectively. This implied that there were differences in the strength of the intermolecular hydrogen-bond interactions in these xerogels, even though they were from the same solvent system. The present data further verified that the substituent groups in molecular skeletons can regulate the stacking of the gelator molecules to self-assemble into ordered structures by distinct intermolecular hydrogen bonding [39].

**Figure 7:**
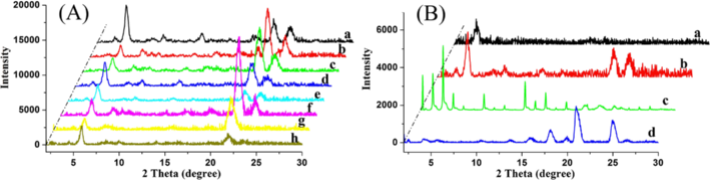
**X-ray diffraction patterns of xerogels. **(**A**) TC16-Azo-Me (**a**, nitrobenzene; **b**, aniline; **c**, acetone; **d**, ethyl acetate; **e**, DMF; **f**, n-propanol; **g**, n-butanol; and **h**, n-pentanol); (**B**) **a**, TC16-Azo; **b**, TC16-Azo-Me; **c**, SC16-Azo; and **d**, SC16-Azo-Me, in DMF.

Furthermore, in order to investigate the orderly stacking of xerogel nanostructures, XRD of all compound xerogels from gels were measured. Firstly, TC16-Azo-Me samples were taken as example, as shown in shown in Figure 7A. The curves for TC16-Azo-Me xerogel samples show similar main peaks in the angle region (2θ values: 5.26°, 7.74°, 21.38°, and 23.12°) corresponding to the *d* values of 1.68, 1.14, 0.42, and 0.38 nm, respectively. The corresponding *d* values of 1.68 and 0.42 nm follow a ratio of 1:1/4, suggesting a lamellar-like structure of the aggregates in the gel [40]. In addition, the XRD data of xerogels of all compounds in DMF were compared, as shown in Figure 7B. Firstly, the curve for TC16-Azo xerogel in DMF shows one weak peak at a 2θ value of 4.36° corresponding to the *d* value of 2.03 nm. As for the curve of SC16-Azo, many peaks were obtained, suggesting a polycrystalline structure. In addition, only a little bit peaks in the low angle range observed in the curve of SC16-Azo-Me, indicating an amorphous state. The XRD results described above demonstrated again that the substituent groups had a great effect on the assembly modes of these compounds.

## Conclusions

Four azobenzene imide derivatives with different substituent groups have been synthesized. Their gelation behaviors in various organic solvents can be regulated by changing alkyl substituent chains and headgroups of azobenzene segment. The substituent groups in azobenzene residue and benzoic acid derivatives can have a profound effect upon the gelation abilities of these studied compounds. More alkyl chains in molecular skeletons in present gelators are favorable for the gelation of organic solvents. Morphological studies revealed that the gelator molecules self-assemble into different aggregates, from wrinkle, lamella, and belt to fiber with the change of solvents. Spectral studies indicated that there existed different H-bond formations between imide groups and conformations of methyl chains, depending on the substituent groups in molecular skeletons. These results afford useful information for the design and development of new versatile low molecular mass organogelators and soft matter.

## Competing interests

The authors declare that they have no competing interests.

## Authors' contributions

TJ carried out the synthesis of compounds and characterization of organogels. YW participated in the analysis and the testing of the nanostructures. QZ and FG supervised this work, helped in the analysis and interpretation of data, and, together with JZ, worked on the drafting and revisions of the manuscript. TJ and QZ conceived of the study and participated in its design and coordination. JZ participated in the design of the study and provided analysis instruments. All authors read and approved the final manuscript.

## Authors' information

TJ and QZ are associate professors. YW is an MD student. FG is a professor and the Dean of the School of Environmental and Chemical Engineering. JZ is a laboratory assistant in Yanshan University.
